# A new anabolic compound, LLP2A-Ale, reserves periodontal bone loss in mice through augmentation of bone formation

**DOI:** 10.1186/s40360-020-00454-x

**Published:** 2020-11-13

**Authors:** Min Jiang, Lixian Liu, Ruiwu Liu, Kit S. Lam, Nancy E. Lane, Wei Yao

**Affiliations:** 1 Department of Internal Medicine, University of California, Davis Medical Center, 4625 2nd Avenue, Sacramento, CA 95817 USA; 2 Shanghai Key Laboratory for Prevention and Treatment of Bone and Joint Diseases, Shanghai Institute of Traumatology and Orthopaedics, Ruijin Hospital, Shanghai Jiao Tong University School of Medicine, 197 Ruijin 2nd Road, Shanghai, 200025 China; 3 Yunan Vocational and Technical College of Agriculture, Kunming, 650031 Yunan China; 4 Department of Biochemistry & Molecular Medicine, University of California Davis, Sacramento, CA 95817 USA

## Abstract

**Background:**

Currently, there are no effective medications to reverse periodontal disease (PD)-induced bone loss. The objective of this study was to test a new anabolic compound, LLP2A-Ale, or with the combination treatment of mesenchymal stromal cell (MSC), in the treatment of bone loss secondary to PD.

**Methods:**

PD was induced in mice by placing a ligature around the second right molar. At one week after disease induction, the mice were treated with placebo, LLP2A-Ale, MSCs, or combination of LLP2A-Ale + MSCs, and euthanized at week 4.

**Results:**

We found that PD induced alveolar bone loss that was associated with reduced bone formation. LLP2A-Ale alone or in combination with MSCs sustained alveolar bone formation and reversed alveolar bone loss. Additionally, PD alone caused systemic inflammation and increased the circulating levels of G-CSF, IP-10, MIP-1a, and MIP2, which were suppressed by LLP2A-Ale +/− MSCs. LLP2A-Ale +/− MSCs increased bone formation at the peripheral skeletal site (distal femur), which was otherwise suppressed by PD.

**Conclusion:**

Our findings indicated that LLP2A-Ale treatment rescued alveolar bone loss caused by PD, primarily by increasing bone formation. LLP2A-Ale also attenuated the circulating levels of a series of inflammatory cytokines and reversed the PD-induced suppression of systemic bone formation.

## Background

Periodontal disease (PD) is characterized by the progressive destruction of tooth-supporting alveolar bone and is mainly caused by chronic inflammation in response to persistent bacterial insult [1]. Currently, there are no effective medications that reverse PD-induced bone loss and regenerate new bone. There is an unmet medical need for nonsurgical therapeutic options to treat bone loss induced by PD. There are limited drug interventions for PD-induced alveolar bone loss that have been focused on reducing bone resorption. The most commonly available antiresorptive agents are bisphosphonates, which may be associated with an increased risk for osteonecrosis [2–4]. Other medications include antibiotics, and gluconate mouthwash, which reduce bacteria in the mouth but do not have direct effects on bone. To this end, we have developed a novel compound, LLP2A-Ale, that has an affinity for both bone (hydroxyapatite tissue) and mesenchymal stromal cells (MSCs). LLP2A-Ale improves the bone homing of both endogenous and exogenous MSCs [5, 6]. Several studies have been conducted to test this approach in animal models of primary osteoporosis, aging, glucocorticoid-induced bone fragility, and fracture healing [5, 7, 8]. Our data, as well as those from others, have confirmed that MSCs have paracrine effects that reduce inflammation and restore angiogenesis and osteogenesis when they are localized within the bone microenvironment [8–12]. LLP2A-Ale is currently in Phase I clinical trial for safety evaluation of this compound in glucocorticoid-induced osteopenia patients, with a further indication of treating osteonecrosis in Phase II. The current study evaluated aimed to evaluate whether LLP2A-Ale, alone or in combination with MSCs, represents a new approach with increased anti-inflammatory activity and bone regeneration, which are essential for the treatment of bone loss and growth of new bone to decrease the risks of tooth loss and dental implants secondary to PD.

## Methods

### Animal protocols

**Fig. 1:**
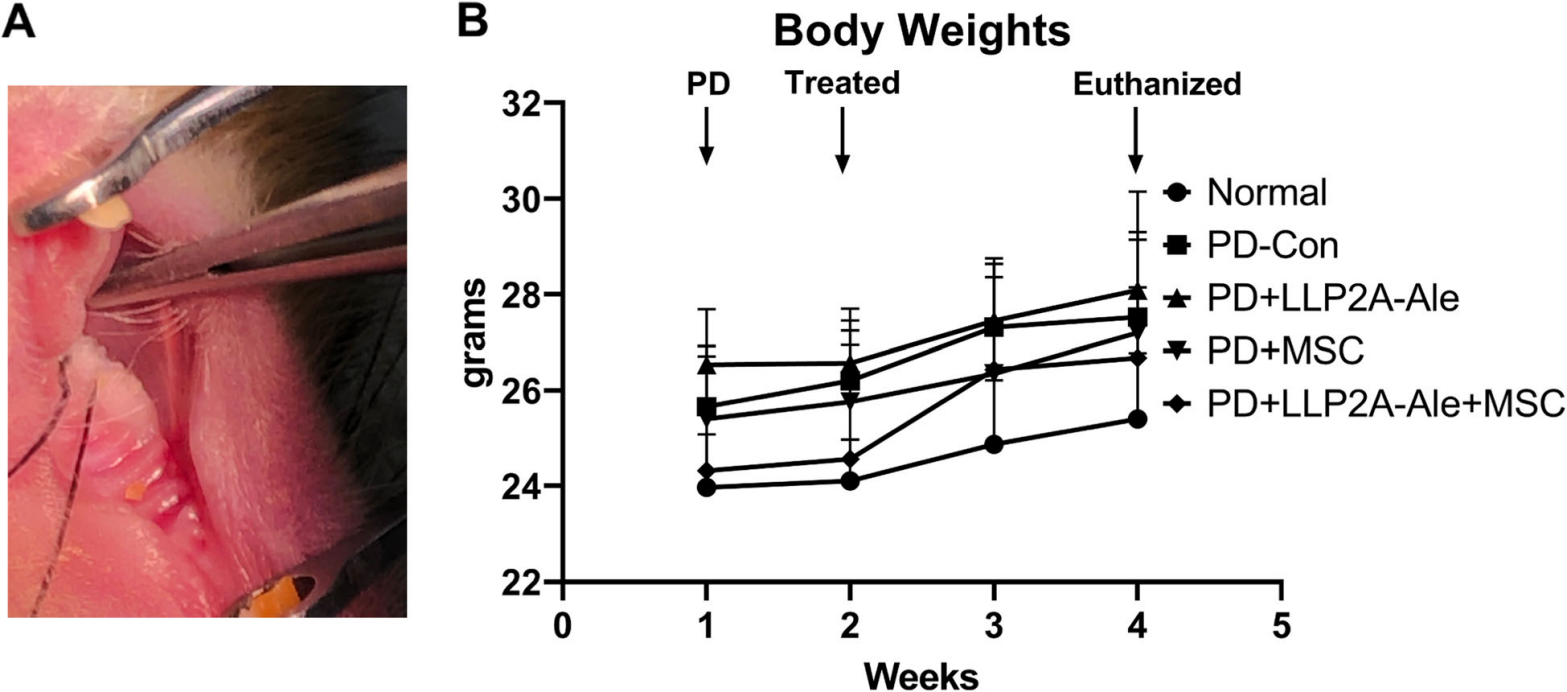
Periodontitis mouse model and general well-being of the mice during the study. **a** A ligature was placed around the right second molar of eight-week-old BALB/C mice at week 1. **b** Treatment started at week 2, and mice were euthanized at week 4. Bodyweight was recorded weekly during the study period

Eight-week-old BALB/C male mice were used in the study (The Jackson Laboratory, Cat#000651). To induce PD, 6–0 silk ligatures (Fine Science Tools, San Diego, CA, USA) were tied around the right 2nd molars with the knot at the palatal surface of the tooth (Fig. 1a). Control mice did not receive ligatures. Since ligand-induced peak bacterial load and apparent periodontal bone loss that was observed between days 5–7 post-ligature [13–15], we initiated the interventions on day 8. All the ligatured mice were randomized into four groups of 8–12 mice each: PD-control (normal saline); PD + LLP2A-Ale (500 μg/kg weekly by i.p. injection); PD + MSCs (Cell Biologics, Chicago, IL, USA; 1 × 10^6^ i.v. on day 8); and PD + LLP2A-Ale (1000 μg/kg) + MSCs (1 × 10^6^ i.v. on day 8). Ligatures were checked weekly and retied if they became loose. One week and one day before sacrifice, animals were injected with 10 mg/kg calcein (Sigma-Aldrich, MO, USA). All mice were euthanized by CO2 overdose at week 4. The animal procedures and euthanization protocol were approved by the Institutional Animal Care and Use Committee at UC Davis.

### Cytokine multiplex assay

The levels of cytokines in serum were measured using a 32-plex mouse immunology cytokine/chemokine kit purchased from Millipore Sigma (Burlington, MA, USA) that included eotaxin/CCL11, G-CSF, GM-CSF, IFN-γ, IL-1α, IL-1β, IL-2, IL-3, IL-4, IL-5, IL-6, IL-7, IL-9, IL-10, IL-12 (p40), IL-12 (p70), IL-13, IL-15, IL-17, IP-10, KC, LIF, LIX, MCP-1, M-CSF, MIG, MIP-1α, MIP-1β, C-X-C motif chemokine ligand 2 (CXCL2, MIP-2), RANTES, TNF-α, and VEGF. The samples were run in duplicate using EMD Millipore LMX200 system (Burlington, MA, USA).

### MicroCT measurements

**Fig. 2:**
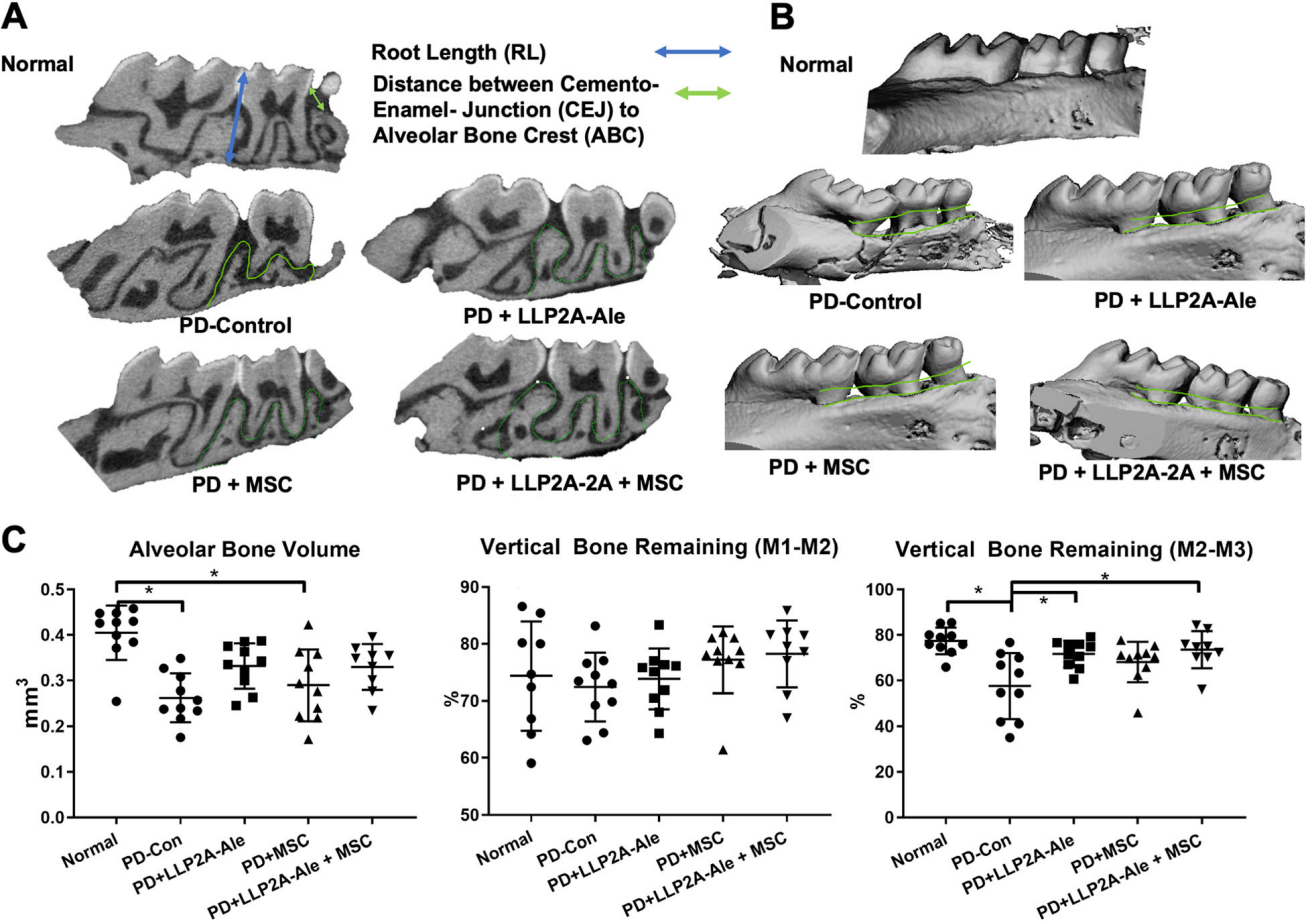
PD induced alveolar bone loss that was reversed by LPP2A-Ale +/− MSCs. **a** Representative two-dimensional sagittal images of the right molars in each group. Blue double-headed arrows illustrate root length. Green double-headed arrows indicate the distance between the cement enamel junction (CEJ) and the alveolar bone crest (ABC). Green curved lines highlight the region of interest for the three-dimensional evaluation of alveolar bone volume. **b** Representative three-dimensional sagittal images of the right molars in each group. **c** Quantitative measurements of alveolar bone volume and the vertical bone remaining between M1-M2 and M2-M3. * *p* < 0.05 for comparisons of the indicated groups

The right maxillae were scanned with a micro-computed tomography system (vivaCT 40; SCANCO Medical, Switzerland) with a resolution of 10.5 μm at 75 kVp. The sagittal plane of the specimens was set parallel to the X-ray beam axis. Two-dimensional measurements of vertical bone were performed in three central slides that included the three molars in the same grayscale image. The linear distance was measured from the junction of the head and shaft of the implant to the alveolar bone crest (ABC) or vertically from the cement enamel junction (CEJ) to the ABC. Measurements were made in the interdental region between the first and second molars (M1-M2) or the second and third molars (M2-M3). We used the methods reported by Part et al. 2007 to calculate the amount of vertical bone remaining [16] (Fig. 2). To assess volumetric bone mass, alveolar bone parameters of bone volume (BV) and bone volume fraction (BV/TV) were derived from manually contour-drawn regions of interest including the alveolar bone between the 1st and 3rd molars, as highlighted in green in Fig. 2a**.** MicroCT evaluations were performed by two calibrated examiners (LXL and WY) who were blinded to group assignment.

### Histology

Following sacrifice, the maxillae were collected, fixed for 48 h in 4% paraformaldehyde and stored in − 80 °C until being dehydrated in 30% sucrose and were embedded in optimal cutting temperature (O.C.T.; Tissue-Tek, Thermo Fisher, Waltham, MA, USA) and cut into serial 8-μm-thick sections. Cryosections were prepared and imaged with a Keyence BZ-X9000 all-in-one Fluorescence Microscope (Itasca, IL, USA) for MSC tracking (*n* = 4/group). Surface-based bone formation was quantitated using BIOQUANT image analysis software (*n* = 4/group) (BIOQUANT Image Analysis Corporation, Nashville, TN, USA) [17].

### Statistical analysis

The results are expressed as the mean ± standard deviation. One-way ANOVA was used to detect significant differences among the groups. When significance was detected, Dunnett’s multiple comparisons test was used to assess pairwise comparisons between groups. A value of *p* < 0.05 was considered to indicate statistical significance. Data were analyzed using GraphPad Prism 8 software (La Jolla, CA, USA).

## Results

### The general well-being of the mice after ligature placement and treatment

A ligature was placed around the right second molar at week 1 in male eight-week-old BALB/C mice (Fig. 1a). Treatment was started at week 2. All the mice gained an average of 5% body weight during the study period. The body weight leveled off in the PD-control group at week 3, while the PD + LLP2A-Ale + MSC group showed a significant increase of 12% body weight at week 4 compared to baseline (Fig. 1b).

### Alveolar bone loss following PD and treatment

The distance from the CEJ to the AC was measured at the buccal root of the first maxillary molar and the second molar and between the palatal root of the second maxillary molar and the third molar. Three weeks after ligature placement around the 2nd molar, there was a significant increase in the CEJ-AE distance between the 2nd-3rd molars, indicating periodontal bone loss (Fig. 2a). Three-dimensional reconstruction of the right maxilla confirmed the difference in periodontal bone height in the ligature-induced PD group compared to the normal, non-ligatured control group and the other treatment groups (Fig. 2b). Volumetrically, ligature placement caused significant alveolar bone loss and reduced the height of the remaining vertical bone (Fig. 2c). PD + MSC monotherapy evoked similar changes as PD alone, while PD + LLP2A-2A with or without combined MSC treatment preserved the alveolar bone mass and vertical bone remaining at M2-M3.

### PD reduced bone formation in the maxilla

**Fig. 3:**
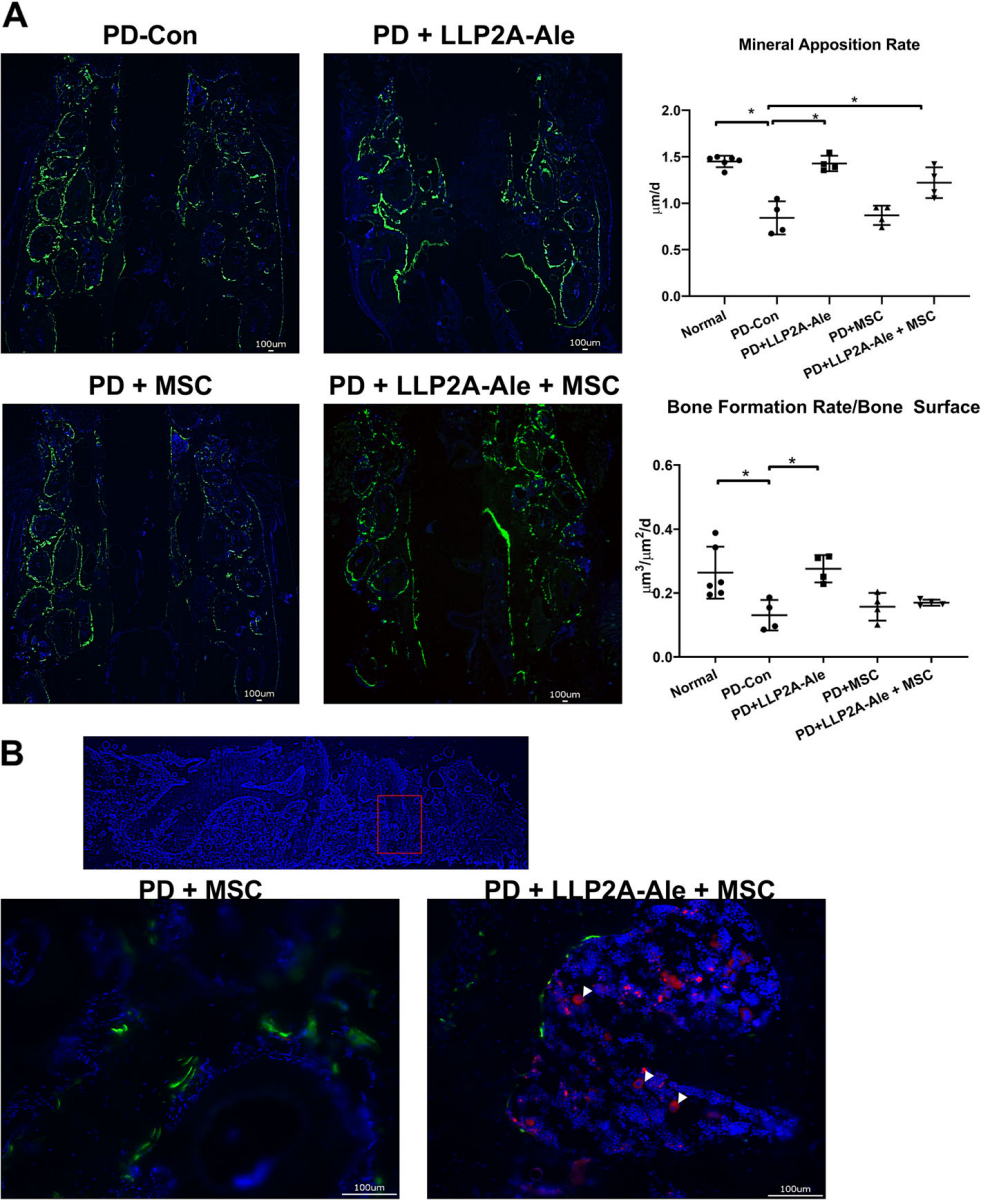
The PD-induced reduction in maxilla bone formation was reversed by LLP2A-Ale +/− MSCs. **a** Representative axial maxilla images in each group. All mice received double calcein labeling before euthanization. The right frames are the enlarged areas indicated in the boxes. White arrowheads indicate some transplanted td-Tomato^+^ MSCs. **b** Quantitative measurements of the mineral apposition rate and surface-based bone formation rate at the right maxilla. * *p* < 0.05 for comparisons of the indicated groups

Calcein-labeled frozen maxilla sections were assessed to measure bone apposition. Axial frozen sections of the right and left maxillae were obtained to analyze surface-based bone formation. A cursory evaluation of maxillae suggested a decrease in the total labeled surface in right maxillae (with a ligature) compared to left maxillae (no ligature) in both the PD-alone and PD + MSC groups (Fig. 3a). Quantitatively, the mineral apposition rate (MAR) was decreased by more than 40% in the PD-alone and PD + MSC groups compared to the normal no ligatured group (*p* < 0.05). The MAR was normalized in both the PD + LLP2A-Ale and PD + LLP2A-Ale + MSC groups, in which it increased by 70 and 45%, respectively, compared to the PD-alone group (*p* < 0.05). The surface-based bone formation rate (BFR/BS) was decreased by more than 50% in the PD-alone as well as in PD + MSC groups compared to the normal non-ligatured group (*p* < 0.05). The BFR/BS returned to normal in both the PD + LLP2A-Ale and PD + LLP2A-Ale + MSC groups. LLP2A-Ale increased BFR/BS by 110% compared to PD alone (*p* < 0.05) (Fig. 3b). Notably, there were many transplanted MSCs (Fig. 3a, white arrowheads) in the PD + LLP2A-Ale + MSC group, but these MSCs were not on the bone surface and did not colocalize with the double-labeled surface.

### Systemic inflammation following PD

**Fig. 4:**
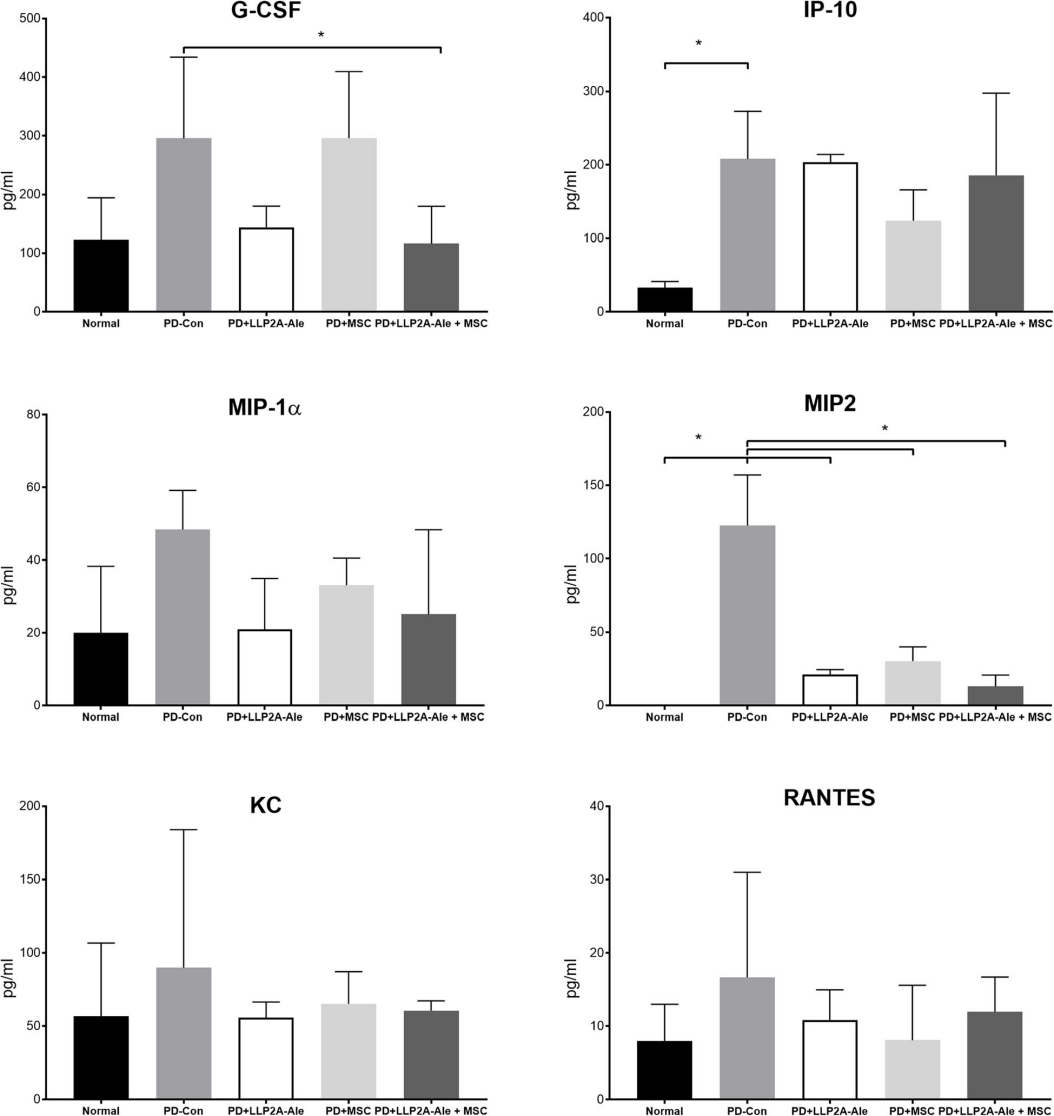
Systemic cytokine/chemokine changes. Cytokine/chemokine levels were measured in the serum at week 4. Detectable cytokines/chemokines are presented. * *p* < 0.05 for comparisons of the indicated groups

To monitor the changes in systemic inflammation following PD, we measured cytokine and chemokine levels in serum after the mice were euthanized. Among the 32 cytokines and chemokines that were measured, most were below the detection limit, except for G-CSF, IP-10, MIP-1α, MIP2, KC, and RANTES. The PD-control group showed significantly increased serum levels of IP-10, and MIP2 (*p* < 0.05 compared to the normal group) and a trend toward higher G-CSF and MIP-1α ES levels (Fig. 4), which were lower in the LLP2A-Ale and LLP2A-Ale + MSC-treated groups.

### Systemic reduction in bone formation following PD

**Fig. 5:**
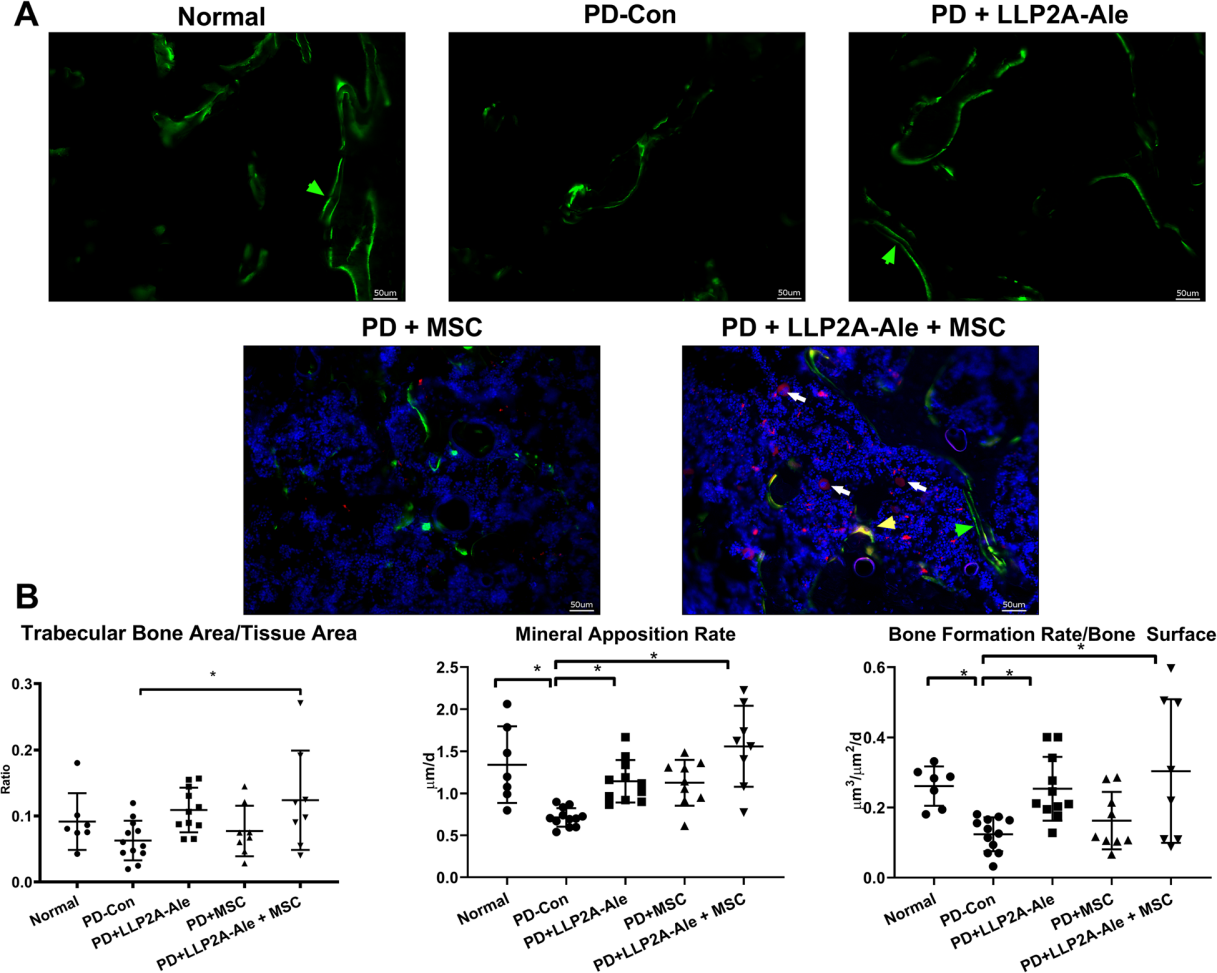
The PD-induced reduction in systemic bone formation was reversed by LLP2A-Ale+/− MSCs. **a** Representative distal femoral metaphysis images in each group. All mice received double calcein labeling before euthanization (green arrows). White arrows indicate some transplanted td-Tomato^+^ MSCs, some of which were positive for calcein and appeared yellow (yellow arrow). **b** Quantitative measurements of the trabecular bone area, mineral apposition rate, and surface-based bone formation rate at the right distal femur. * *p* < 0.05 for comparisons of the indicated groups

To assess whether PD is associated with systemic changes in bone turnover, we obtained frontal sections of the right femur metaphysis (DFM) and lumbar vertebral body for measurements of peripheral skeletal bone mass and bone formation. We report the DFM results here. PD decreased trabecular bone area by 31% compared to the control (no ligature), and the trabecular bone area was increased 70% by LLP2A-Ale and 97% by PD + LLP2A + MSC (*p* < 0.05) compared to PD alone (Fig. 5). There was a significant reduction in the double-labeled surface in the PD and PD + MSC groups, suggesting reduced osteoblast activity (Fig. 5a, green arrows). Quantitatively, the MAR was decreased by nearly 50% in the PD-alone as well as the PD + MSC groups compared to the normal non-ligatured group. The MAR was rescued to a normal level in both the PD + LLP2A-Ale and PD + LLP2A-Ale + MSC groups, in which it was increased by 55 and 110%, respectively, compared to the PD-alone group (*p* < 0.05). The BFR/BS was decreased by more than 50% in the PD-alone and PD + MSC groups compared to the normal non-ligatured group. The BFR/BS returned to normal in both the PD + LLP2A-Ale and PD + LLP2A-Ale + MSC groups, in which it was increased by 106 and 167%, respectively, compared to the PD-alone group (*p* < 0.05) (Fig. 5b). There were many transplanted MSCs (Fig. 5a, white arrows) in the bone marrow in PD + LLP2A-Ale + MSC-treated mice, and some of the transplanted MSCs turned yellow and were double-positive for red (tdTomato) and green (calcein) (Fig. 5a, yellow arrow), suggesting active mineral apposition in these transplanted MSCs.

## Discussion

Periodontal disease resulting from chronic dental infection can cause periodontal bone loss or, in the worst case, tooth loss. The current investigation evaluated anabolic treatment with a novel stem cell bone-targeting drug, LLP2A-Ale, with or without exogenous MSCs to promote bone formation and treat alveolar bone loss associated with PD.

Ligature-induced periodontitis is a well-established mouse model that recapitulates the pathogenesis of human periodontal disease, including bacteria film formation, host immune response stimulation, and alveolar bone loss. Ligand-induced bacterial load and peak periodontal bone loss were observed between days 5–8 post-ligature, which might sustain for 2–3 weeks, or resulted in tooth loss [13–15]. We started treatment on day 8, representing an intervention protocol. In this study, we provided radiographic and histologic evidence that PD induced alveolar bone loss and suppressed bone formation. Treatment with LLP2A-Ale, alone or in combination with MSCs, rescued alveolar bone loss by augmenting bone formation, supporting the use of an anabolic approach to sustain bone formation for the treatment of bone loss associated with PD. Importantly, PD was also associated with systemic changes in cytokines and chemokines and induced changes in peripheral bone formation and bone mass, resembling a systemic inflammatory disease. LLP2A-Ale monotherapy and LLP2A-Ale + MSCs were equally effective at attenuating the PD-induced suppression of bone formation, sustaining alveolar bone mass, and increasing systemic bone formation.

PD is characterized by both inflammation and bone loss [18]. Current medical treatments for PD include flap surgery/root planning, antibacterial oral rinses or antibiotics to control infection, and antiresorptive treatments to control periodontitis. Some non-surgical therapeutics, such as the adjunctive use of probiotics (Clinical study identifier NCT04069611) and Omega-3 polyunsaturated fatty acids (Clinical study identifier NCT04477395) are being evaluated for their efficacies in treating PD. Taurolidine gel was shown to have antimicrobial effects and useful for periodontal therapy [19]. Other experimental drugs, such as chlorhexidine, tetracycline/hydrochloride, locale application of statins, were used as adjunctive treatments to scaling and root planning and were found to have antimicrobial, anti-inflammatory, and modulated bone remodeling process in a rat model of PD [20–23].

Other medications for osteoporosis, such as bisphosphonates (BPs), are used to treat bone loss resulting from periodontitis. BPs are shown to inhibit alveolar bone resorption [24, 25]. However, chronic or high-dose BP treatment is associated with a higher risk for the development of osteonecrosis of shown to inhibit alveolar bone resorption [24, 25]. However, chronic or high-dose BP treatment is associated with a higher risk for the development of osteonecrosis of the jaw in animals [4, 26–28] and humans [3, 4, 29, 30]. Other medications that affect bone turnover, such as a monoclonal antibody against receptor activator of NF-κB ligand (RANKL), inhibits bone resorption [31]. An antibody against high mobility group box 1 (HMGB1), a nonhistone DNA-binding protein that is secreted into the extracellular matrix in response to inflammation, suppresses the progression of periodontitis through an antiresorptive mechanism [32]. Antiresorptive agents such as BPs are also known for their antiangiogenic actions that potentially are a risk factor for the development of osteonecrosis [33, 34]. Anabolic treatments, such as teriparatide (human PTH (1–34), which effective against osteoporosis, may also help treat bone loss in both rat and mouse models of PD [35, 36]. Interestingly, these previous studies reported antiresorptive and anti-inflammatory mechanisms for daily PTH injections instead of the well-known bone growth effects usually observed for PTH [35, 36]. In one clinical trial, the application of PTH combined with periodontal surgery was superior to periodontal surgery alone at repairing localized bone defects [37]. PTH was shown to enhance alveolar bone formation in conjunction with bone grafts in animal studies [38, 39]. Biweekly injections of a new anabolic agent, a monoclonal antibody against sclerostin, for 6 weeks increased periodontal bone formation and alveolar bone in a rat model of PD [40]. Taken together, the data indicate that bone anabolic agents, rather than antiresorptive agents, may be more beneficial for the treatment of periodontal bone loss than anti-resorptive treatment. On the other hand, our previous studies with LLP2A-Ale have found enhanced angiogenesis and blood vessel density in bone [7, 8]. The ability of LLP2A-Ale to stimulate both angiogenesis and bone formation suggests that this agent has considerable potential as a treatment of periodontal bone loss.

Cytokines have an essential role in the pathogenesis of PD. The host response can attenuate periodontal bone loss, mostly through anti-resorptive mechanisms [41, 42]. In PD tissue, abundant neutrophils are localized in connective tissue [43, 44]. In the initial steps of periodontal disease, gingival epithelial cells defend against bacterial infection by secreting cytokines such as interleukin (IL)-8, granulocyte-macrophage colony-stimulating factor (GM-CSF), and monocyte chemotactic protein (MCP-1) from gingival tissues to induce the migration of immune cells into inflammation sites [45–47]. These various inflammatory cytokines, while defend against bacterial infections, may indirectly stimulate osteoclastogenesis and bone resorption. Other chemokines, such as CXCL10, CXCL12, CXCL13, and CCL5, may affect osteoblast precursors or osteoblasts and therefore bone formation [48–52]. When the inflammation is limited to the subepithelial space, it induces damages to the underlying bone through recruitment of osteoclast precursors and induce bone resorption, and may not result in no net bone loss [53, 54]. If the inflammatory infiltrate persists near the bone, the bone formation will be uncoupled due to the inhibitory effects of cytokines on osteoblasts [18]. However, inflammation is complicated for PD; while low-level inflammation may be protective against the constant bacterial insult, high levels of key inflammatory factors, such as IL-1, TNF-α, and IL-17R, can result in more severe disease [55–57], and IL-10 may protect against periodontal bone loss [58]. Alendronate was used in LLP2A-Ale to achieve bone-targeted effects [5, 6], however, we have not observed anti-resorptive effects in other published studies [5–8]. Additionally, we initiated treatment with LLP2A-Ale with and without MSCs when the periodontal bone loss was established [13, 14], and we chose to focus on evaluating the ability of LLP2A-Ale to augment new bone formation. Three weeks after the ligature was placed to induce PD, the surrounding alveolar bone had reduced the mineral apposition rate, corresponding to reduced osteoblast activity, contributing to the observed alveolar bone loss.

It is among the shortcoming of our report that we did not measure pocket formation, a surrogate for inflammation and bone loss, and the local inflammatory cells infiltration in the periodontal tissues. We measured the systemic serum cytokine levels and found that inflammation assocaited with the PD is not localized to the dental area but was associated with increased systemic levels of interferon-gamma inducible protein 10 (IP-10), macrophage inflammatory protein-1alpha (MIP-1α), RANKL, G-CSF, and macrophage inflammatory protein 2 (MIP2, CXCL2). Higher expression levels of IP-10, RANTE, MCP-1, and MIP2α were found in gingival biopsies from PD patients [59, 60], and a sustained increase in the concentration of both MIP-1a and MCP-1 was associated with increasing severity of PD [61]. Moreover, investigators have reported that PD may trigger a systemic immune response associated with chronic disorders, including cardiovascular disease, obesity/type 2 diabetes, and rheumatoid arthritis [62]. In particular, we found that PD increased the circulating levels of G-CSF and MIP2 (CXCL2), both of which attenuated osteoblast differentiation [63–65]; this result was consistent with our observation of a reduced mineral apposition rate locally in the oral region and systemically, which ultimately resulted in localized and generalized bone loss. Both the elevated serum levels of G-CSF and MIP2 levels were suppressed by LLP2A-Ale treatment, thus maintaining periodontal bone formation and bone mass. LLP2A-Ale + MSCs increased systemic bone formation and bone mass, which was consistent with our previous observations in other models of bone disease [5–8]. However, in this PD model of inflammation and bone loss, we did not see an additive effect of MSC transplantation for alveolar bone formation during the treatment of periodontal bone loss despite the observation that transplanted MSCs were homed to the periodontal regions. MSCs are known to have paracrine effects that support anti-inflammatory activity, angiogenesis, and osteogenesis within the bone microenvironment [8], but we have yet to evaluate the fate of these transplanted MSCs engrafted in alveolar bone and ascertain whether they have an anti-inflammatory or pro-angiogenetic effect in the longer term. Rather than using bone-marrow-derived MSCs, other sources of MSCs, especially the oral stem cells that are derived from the periodontal ligament, dental pulp, exfoliated deciduous teeth, dental follicle, and gingival et al., may offer superior regenerative effects to support periodontal repair or regeneration [66–68]. Periodontal ligament -derived stem cells were used to treat PD in humans (clinical trial identifier: NCT02523651). Apart from oral stem cells, other regenerative approaches such as the use of biomaterials, by themselves or used as scaffolds to load cells or drugs, extracellular vesicles, hold promising therapeutic future to repair and grow bone [69, 70]. Nevertheless, additional studies are needed to determine the beneficial effects of using dental-derived stem cells and LLP2A-Ale for PD-induced bone loss. Perhaps combination treatments of dental-derived MSC and an anabolic agent would be ideal to treat more a challenge degenerative dental diseases, such as repairing osteonecrosis of the jaw, to reduce inflammation, restore angiogenesis, and induce bone regeneration.

In conclusion, LLP2A-Ale treatment stimulates alveolar bone formation and reverses alveolar bone loss induced by ligature induced PD. LLP2A-Ale also reduces PD-induced circulating inflammatory proteins, especially, G-CSF and MIP2. Treatment with the combination of MSCs and LLP2A further increases peripheral skeletal bone formation and bone mass but has no additive effect in terms of further increasing alveolar bone formation. Monotherapy with weekly injections of LLP2A-Ale is a novel therapeutic option for the treatment of bone loss associated with periodontitis.

## Data Availability

Materials described in the manuscript, including all relevant raw data, were archived in UC Davis share drive, and will be available to any scientist wising to use them for non-commercial purposes by sending a request to the corresponding author.

## Abbreviations

PD: Periodontal disease
MSC: Mesenchymal stromal cell
i.p.: Intraperitoneal
i.v.: Intravenous
CXCL2 (MIP2): C-X-C motif chemokine ligand 2
ABC: Alveolar bone crest
CEJ: Cement enamel junction
AC: Alveolar crest
BV/TV: Bone volume to the tissue volume fraction
MAR: Mineral apposition rate
BFR/BS: Surface-based bone formation rate
DFM: Distal femur metaphysis
RANKL: Receptor activator of NF-κB ligand
HMGB1: High mobility group box 1
IL: Interleukin
GM-CSF: Granulocyte-macrophage colony-stimulating factor
MCP-1: Monocyte chemotactic protein-1
